# Reliability of Ankle–Foot Complex Isokinetic Strength Assessment Using the Isomed 2000 Dynamometer

**DOI:** 10.3390/medicina54030043

**Published:** 2018-06-04

**Authors:** Zuzana Gonosova, Petr Linduska, Lucia Bizovska, Zdenek Svoboda

**Affiliations:** Department of Natural Sciences in Kinanthropology, Faculty of Physical Culture, Palacky University, 771 11 Olomouc, Czech Republic; petr.linduska@upol.cz (P.L.); lucia.bizovska@gmail.com (L.B.); zdenek.svoboda@upol.cz (Z.S.)

**Keywords:** plantar flexors, dorsal flexors, ankle invertors, ankle evertors, reproducibility

## Abstract

For quantifying muscle strength in clinical and research practice, establishing the reliability of measurements, specifically to the procedures used, is essential for credible findings. The objective was to establish the reliability of isokinetic measurement of ankle plantar and dorsal flexors (PF/DF) and invertors and evertors (INV/EV) on an IsoMed 2000 dynamometer. Twenty healthy subjects (10 males, 10 females, mean age: 23.1 ± 3.1 years) completed an isokinetic measurement session. The intraclass correlation coefficient (ICC) and standard error of measurement were assessed for peak torque and work of ankle PF/DF (concentric and eccentric) and INV/EV (concentric) for the preferred and nonpreferred limb. Standardized isokinetic measurements of reciprocal PF/DF and INV/EV muscle actions were associated with ICC ranging from 0.77 to 0.98 for the majority of observed parameters. The exception was work in the eccentric mode in the ankle DF and peak torque in the concentric mode in the ankle INV on the preferred limb, where ICC ranged from 0.64 to 0.71. The IsoMed 2000 isokinetic dynamometer can be reliably employed in future studies for reciprocal ankle PF/DF and INV/EV assessment in healthy adult subjects after implementation of a familiarization session.

## 1. Introduction

The ankle is the joint of the lower extremity in close proximity to the body’s base of support and therefore ankle muscles play an integral role in gait and balance tasks [1]. In view of the complexity of ankle joint muscles’ involvement in common daily and sport activities [2,3], the reliability of the performance of the plantar flexors (PF) and dorsal flexors (DF), as well as invertors (INV) and evertors (EV), is of interest. For quantifying muscle strength characteristics in clinical and research practice, the reliability of the measurements must be determined for eligible and justified usage of any device, method, or testing protocol [4].

In muscle strength assessment, isokinetic dynamometry is considered the “gold standard” [5]. The reliability of various isokinetic testing protocols for both ankle-related movements, i.e., PF/DF and INV/EV, has been established for different brands of available isokinetic dynamometers [6,7,8,9]. The reliability represented by the intraclass correlation coefficient (ICC) values in the angular velocities in the range of 30–120°/s in the concentric mode of muscle action described in healthy adult subjects for peak torque (PT) values has varied as 0.53–0.80 in PF and 0.20–0.91 in DF [6,10,11], and 0.55–0.96 in INV and 0.54–0.94 in EV [7,8,12]. Furthermore, although eccentric muscle strength is essential to the natural physiological functioning of muscles [13] and has also been studied with regard to ankle instability and ankle injuries [3,14], the reliability of the eccentric mode of action has been considered only by Möller et al. [6], who reported the ICC as 0.69–0.76 for PF and as 0.60–0.95 for DF in a healthy adult population, with a higher reliability for eccentric measurements than for concentric [6]. The studies’ results are, however, difficult to interpret and compare due to differences in testing protocols (e.g., testing position and stabilization, testing velocities and modes of muscle action, passive or active mode of testing, gravitational correction application, testing bare feet or with shoes, etc.) [5,7,15,16].

Furthermore, reliability reports are specific to the procedures used and should not be generalized to other devices, testing protocols, muscle groups, or specific populations such as elderly or children [4,16,17,18]. To the best of our knowledge, there is only one study on the IsoMed 2000 isokinetic dynamometer that reported reliability for ankle testing specifically for PT of ankle PF (ICC = 0.79); however, the study was conducted on children and in static muscle action [19]. To the best of our knowledge, no reliability study describing the isokinetic procedures used to collect data at the ankle–foot complex on the IsoMed 2000 dynamometer has been published. Hence, the purpose of the study was to determine the reliability of measurements on the IsoMed 2000 isokinetic dynamometer for (a) concentric and eccentric plantar and dorsal flexion and (b) concentric ankle inversion and eversion.

## 2. Material and Methods

### 2.1. Sample

The testing sample consisted of 20 adult healthy participants—10 males and 10 females (age: 23.1 ± 3.1 years, height: 176.9 ± 6.7 cm, weight: 71.9 ± 8.7 kg). The participants reported neither neuromuscular nor musculoskeletal injury of the lower extremities in the previous two years and were without previous isokinetic testing experience.

Written statements of informed consent were provided by all participants, and the testing protocol was approved by the Institutional Ethics Committee under identification code 3/2017 in accordance with the ethical standards of the Declaration of Helsinki (1983). Subjects were asked to maintain their regular physical activity during the study period without weight training of their lower extremities. Written informed consent to use pictures was obtained from the participant.

### 2.2. Testing Procedures

Three widely used tests—kicking a ball, crossing over an obstacle, and stepping forward after a push to the back—were used for lower limb lateral preference determination [20]. In all cases, the preferred lower limb was identified as the limb taking action (kicking, stepping over, stepping forward) while the supporting one was marked as a nonpreferred limb. For further analysis, the preferred lower limb was the one scored in at least two tests.

The isokinetic strength of the ankle muscles was measured using the isokinetic dynamometer IsoMed 2000 (D. & R. Ferstl GmbH, Hemau, Germany). Participants completed two measurement sessions: the first represented the very first experience with isokinetic testing (familiarization), and the second represented outcomes after familiarization (real testing). There were seven days between the testing sessions, and both tests were conducted at the same time of day. Each separate visit consisted of two types of isokinetic muscle strength testing: (a) ankle PF and DF and (b) ankle INV and EV. The same examiner with experience in performing isokinetic testing with IsoMed 2000 tested all subjects in both testing sessions. During both sessions, participants wore sportswear and sport footwear of their own choice.

A nonspecific warmup under the supervision of the examiner was performed before the testing. It consisted of 5 min of cycling on a stationary Kettler ergometer (Heinz Kettler GmbH and Co. KG, Ense-Parsit, Germany) at a submaximal intensity (1.5 W/kg of body weight at a pedal rate of 70–80 rpm) followed by 10 min of dynamic stretching exercises for lower limbs. The exercises were selected from Verstegen and Williams [21] as follows: internal and external rotation in the hip, knee and ankle joint, ballistic movement in the anterior–posterior direction in the hip joint with relaxed lower limb, handwalk, lunge stretches, lateral lunges, squats, standing plantar/dorsal flexions and inversion/eversion movements at the ankle joint, calf raises and vertical jump exercises (rope jumps, jumping jacks). There were 6–8 repetitions per stretching exercise and 30 repetitions per jump exercise. Immediately after the nonspecific warmup, participants were positioned and fixed on the adjustable isokinetic dynamometer in agreement with the manufacturer recommendations, as described below. All individual settings were recorded during familiarization and reused during the second session.

#### 2.2.1. Positioning for the Ankle PF and DF

**Figure 1:**
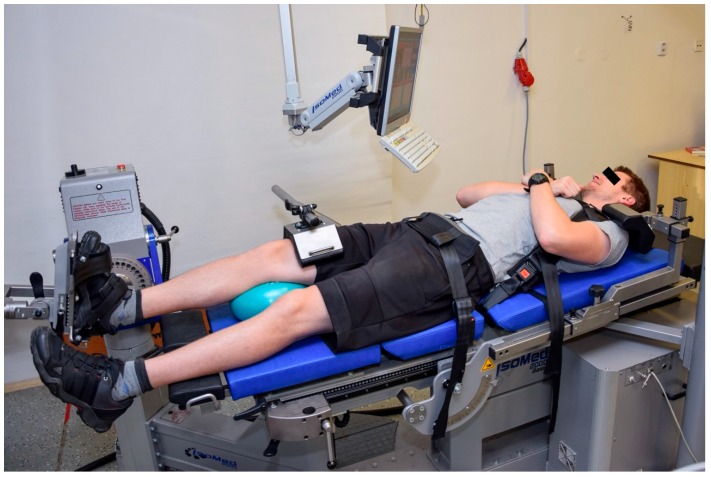
Positioning for the ankle plantar and dorsal flexors testing.

The participants laid supine on the dynamometer seat with their hips and knees in full extension. The foot was placed on the foot adapter connected to the head of a dynamometer and fixed with two Velcro straps. The axis of rotation of the lever arm was aligned with the axis of rotation of the lateral malleolus. The waist and thigh of the tested leg were fixed by straps, and an overball was placed underneath the knee of the tested leg to prevent overextension of the knee and for the subject’s comfort. The shoulders were fixed in the ventral–dorsal and cranial–caudal direction by shoulder straps and pads. The participants were asked to cross their arms in front of the chest. After fixation, a static gravitational correction was applied to negate the influence of the gravity-effect torque on the test data. Figure 1 shows the testing position described above.

#### 2.2.2. Positioning for the Ankle INV and EV

**Figure 2:**
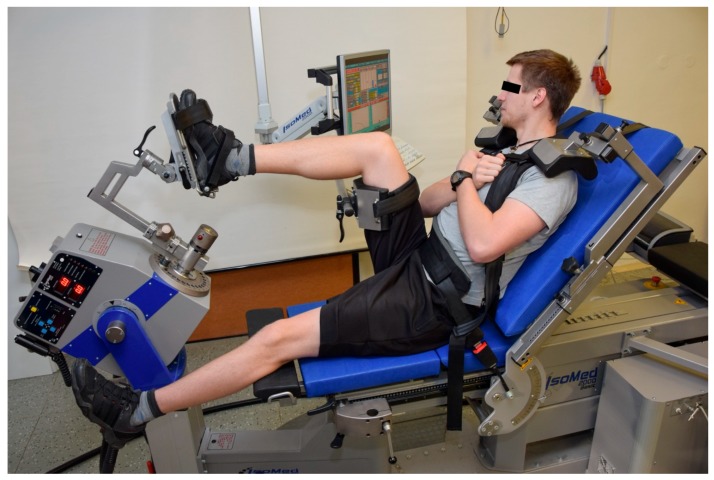
Positioning for the ankle invertors and evertors testing.

The participants were seated on the dynamometer seat with the hip joint at about 80° and knee at 110° (180° = full knee and hip extension), so the shin was positioned horizontally to the ground. The foot was placed on the foot adapter with an ankle angle in 10° of plantar flexion (0° = neutral position of the talocrural joint) and fixed with two Velcro straps. A handheld goniometer was used to set the angles. The hip and knee joint angles were adjusted by changing the distances between the chair and the foot adapter and the height of the support adapter over the thigh. The fixation and arm position were analogous to positioning for testing the ankle PF and DF, as described earlier. Figure 2 shows the testing position described above.

#### 2.2.3. Testing Protocol

Isokinetic testing was conducted at angular velocities of 30°/s and 120°/s in concentric/concentric and eccentric/eccentric reciprocal action for the ankle PF/DF, and at an angular velocity of 30°/s in concentric/concentric reciprocal action for the ankle INV/EV. The lower velocity of 30°/s was chosen because it is referred to a maximal voluntary contraction as an indicator of maximal strength [5] and is the most frequently used velocity in previous ankle-related studies [6,8,10,11,12]. Furthermore, functional tests that resemble activities of daily living are associated with higher angular velocities, such as 120°/s as a velocity of ankle dorsiflexion in the normal gait cycle [22]; therefore, this velocity was also implemented for ankle PF/DF movement. The reliability of the measurements from subjects who began testing at a higher velocity was reported to be much lower compared to when the velocity was lower [17], so we tested all subjects for PF/DF in this study at a lower velocity first in accordance with Hölmback et al. [11]. No preload was set for concentric actions; however, there was a preload of 10 Nm for eccentric action due to difficulties the tested subjects had when performing the test during preliminary testing without any preload (i.e., use a full range of motion during reciprocal eccentric-to-eccentric movement in an active mode of testing). Moreover, a threshold force requirement was implemented in the testing protocol in an attempt to minimize possible force oscillation of varying amplitude in the moment-angular position curve that might occur especially during eccentric muscle action [5]. Prior to each test, all subjects performed three to five submaximal practice trials in tested movements, angular velocities, and muscle actions as a specific warmup and to become acquainted with the requirements of the test. Afterward, tests of three (30°/s) or five (120°/s) maximal nonconsecutive efforts were conducted with a rest interval of 10 s between individual repetitions, 90 s between angular velocities and modes (ankle PF and DF), and 2 min between lower extremities. The three repetitions were chosen because the number of three is commonly used at the velocity of 30°/s [6,8,11,18]. However, in the preliminary testing preceding the current study when a higher testing velocity of 120°/s was adopted, further increases in PT were observed (i.e., in the fourth or fifth repetition). Therefore, five repetitions were chosen for testing at a velocity of 120°/s. A similar approach was used in the studies by Kaminski and Dover [12] and Wennerberg [18]. Each subject was instructed to exert the maximal effort as hard and as fast as possible throughout the whole range of motion [9]. The test for the ankle PF and DF was initiated with subjects in 15° of dorsiflexion with the initial movement toward 35° of plantar flexion (0° = neutral position of talocrural joint). The test for the ankle INV and EV was initiated in 25° of ankle eversion with the initial movement toward 20° of ankle inversion (0° = neutral position of subtalar joint). The participants were notified by a verbal countdown and were provided with strong verbal encouragement and visual real-time feedback to ensure their maximal effort. After testing one lower limb, testing of the other lower limb followed with the same procedure, during which individual settings were automatically activated, rechecked, and adjusted if necessary. The order of the tested movements and limbs was randomized.

### 2.3. Data Processing and Statistical Analysis

For data recording and reduction, the manufacturer’s computer software IsoMed Analyze V.1.0.5 (D. & R. Ferstl GmbH, Hemau, Germany) was utilized. Two isokinetic variables were extracted for each movement (PF/DF, INV/EV), muscle action (concentric, eccentric), and angular velocity (30°/s and 120°/s, depending on the movement): PT and work (W). Due to technical errors, one subject was excluded from analysis of PF/DF at 30°/s in concentric mode and 120°/s in eccentric mode and two subjects were excluded from analysis of PF/DF at 120°/s in concentric mode.

For every analysis, the preferred and nonpreferred limbs were considered separately, and then the mean of the variables obtained for both limbs was computed and subjected to further analysis.

A Kolmogorov–Smirnov test confirmed a normal data distribution of all variables. Statistical analysis was performed in Statistica (v. 12, StatSoft, Inc., Tulsa, OK, USA) and MATLAB (R2016b, MathWorks, Inc., Natick, MA, USA) at a significance level of 0.05.

As a relative reliability measure, ICC(2,1) absolute agreement was used. The within-session reliability was assessed by ICC(2,1) computed from either three or five trials (depending on the movement) recorded during the testing session. Furthermore, the standard error of measurement (SEM) was computed from a total standard deviation (SD) and ICC(2,1) as follows: SEM = SD √(1 − ICC(2,1)). The relative SEM (SEM%) was then computed by dividing the SEM value by the grand mean (X) as follows: SEM% = (SEM/X)∙100%.

## 3. Results

Mean and standard deviation (SD) for ankle-related movements and reliability results (ICC, SEM values) are displayed in Table 1, Table 2, Table 3, Table 4 and Table 5.

For concentric PT of ankle PF and DF the ICC values ranged from 0.85 to 0.97 and the SEM values from 4.0% to 10.3% (1.2 to 7.1 Nm) (Table 1 and Table 2); for eccentric PT the ICC values ranged from 0.79 to 0.98 and the SEM values from 3.0% to 14.1% (1.6 to 25.1 Nm) (Table 3 and Table 4). For concentric W of ankle PF and DF the ICC values ranged from 0.86 to 0.94 and the SEM values from 4.7% to 12.4% (0.9 to 4.6 Nm) (Table 1 and Table 2); for eccentric W the ICC values ranged from 0.71 to 0.96 and the SEM values from 6.8% to 15.2% (2.2 to 8.5 Nm) (Table 3 and Table 4).

The ICC values for ankle INV and EV ranged from 0.64 to 0.94 and the SEM values from 6.1% to 14.5% (1.6 to 3.3 Nm) (Table 5).

## 4. Discussion

Establishing the reliability of the measurements is crucial, because a device that does not yield consistent isokinetic measurements is not relevant without referring to accurate results. In other words, higher reliability of the measurements attained invariably strengthens the degree of credibility of the research findings [4]. This study was conducted to establish the reliability of ankle–foot complex measurements using the IsoMed 2000 isokinetic dynamometer.

For all the movements (ankle PF, DF, INV, EV), velocities (30 and 120°/s), both modes of muscle action (concentric and eccentric) and both limbs (preferred and nonpreferred) the ICC values in our study ranged from 0.77 to 0.98. However, lower ICC values were found on the preferred limb in concentric PT in ankle INV (ICC = 0.64) and in eccentric W in ankle DF at a velocity of 120°/s (ICC = 0.71). This indicates the incoherence in PT production, leading to an assumption that these movements might deserve more attention during a familiarization with the testing conditions. This might be as a consequence of strength-related adaptation tendencies of daily loading associated with functional preferences of the lower limb (initial or support function) in healthy adult subjects [6].

A pattern of slightly higher ICC can be observed in our study in concentric PT for ankle DF (0.94–0.97) in comparison with ankle PF (0.85–0.96). This is contrary to findings observed by Wennerberg [18], who reported no specific pattern as to which motion of the ankle (PF or DF) yielded the highest within-session reliability scores in PT at the same velocities of 30°/s and 120°/s that were used in the present study (PF r = 0.68–0.78; DF r = 0.67–0.79). Despite different statistical analysis in our study (ICC) and the study by Wennerberg [18] (Pearson r values), we can notice a tendency of higher within-session reliability in our study. We assume that the higher reliability of the present results could be explained by an implementation of familiarization session prior to testing.

With regard to testing velocities, a tendency of lower ICC values with increasing velocity for ankle PF and DF can be observed in our study in concentric (PT at 30°/s as 0.93–0.97 vs. PT at 120°/s as 0.85–0.95) as well as eccentric (PT at 30°/s as 0.89–0.98 vs. PT at 120°/s as 0.79–0.95) mode of muscle action. Higher velocities actually require higher rates of torque development [23] and therefore allow for greater variability in performance evaluation, likely due to neural factors [24]. This is in agreement with the study by Hölmback et al. [11] who assessed test-retest reliability for ankle DF for PT at a velocity of 30°/s (ICC = 0.91) and 120°/s (ICC = 0.78). Higher ICC values for ankle DF at the velocity of 30°/s (ICC = 0.79) in comparison with 120°/s (ICC = 0.67) were also detected in the study by Wennerberg [18]. When compared to ICC values of ankle PF with regard to testing velocities in other studies [10,11,18], no clear conclusions could be drawn. In the study by Hölmback et al. [11], only ankle DF were assessed. On the other hand, Wennerberg [18] declared higher ICC for higher velocity. Lastly, Woodson et al. [10] found equal ICC values for both velocities. Not only inconsistencies in the findings, but more importantly a different testing position in these studies with knee flexion angle would be a more relevant reason why it is not possible to conclude that ICC values for ankle PF decrease with increasing testing velocities, as was observed for ankle DF. Different muscle groups or muscle fibers are involved at different joint angles, especially regarding ankle PF [25]. PT in ankle PF in a position with flexed knee is predominantly affected by m. soleus whereas m. gastrocnemius, due to its biarticular action, influences PT production in a position with an extended knee [25].

To the best of our knowledge, only one study on the reliability of ankle PF and DF conducted in eccentric mode of muscle action exists [6]. Higher test-retest ICC values for PT (30°/s) in a position with extended knee were observed for eccentric rather than concentric mode for ankle DF as 0.60–0.71 and 0.20–0.49, respectively [6]. Ankle PF did not evince any specific tendency [6]. In our study, comparable ICC values were found for the modes of muscle action at a velocity of 30°/s. The reason for different findings in the magnitude of ICC between modes of muscle action in the study by Möller et al. [6] and ours might be a different sequence of muscle group involvement in the tested movements—concentric action of DF followed by eccentric action of DF [6] in comparison with eccentric action of DF followed by eccentric action of PF (our study). The first action of the muscle group might affect the subsequent action of the same muscle group in a reciprocal movement. At a higher velocity of 120°/s, a tendency of higher ICC values for PT can be recognized for ankle DF in concentric mode (than eccentric) and vice versa for ankle PF in eccentric mode (than concentric). 

In our study, we observed higher ICC values for ankle PF/DF (0.71–0.98) in comparison with ankle INV/EV (0.64–0.94). The greater degree of variability of ankle-related measurements in the medio–lateral direction (INV/EV) compared to the anterior–posterior direction (PF/DF) was also reported earlier, particularly for EV [9,12,26]. The explanation could arise from a more complicated movement pattern during EV and more natural, functional daily activity, represented by PF/DF, in comparison with INV/EV [9]. Our findings on ankle INV/EV are not in a full agreement with previously reported findings [9,12,26]. We observed slightly higher ICC values for ankle EV than for ankle INV. Although variables describing ankle INV on the nonpreferred limb were approaching the values of ICC similar to those of ankle EV; ankle INV on the preferred limb evinced lower ICC values. The inconsistency of our findings with those reported earlier might be caused by different testing positions and/or inclusion of higher testing velocities in studies of other authors (60°/s–160°/s) that might influence the moment production at the ankle joint [9,26]. Although a similar testing position for INV/EV was used in a study by Kaminski and Dover [12], no stabilization of the upper body was used, as opposed to our study. Stabilization of the body prevents extraneous body movements and the involvement of other muscles in the tested movement [15]. Furthermore, suboptimal securing of the subject might lead to higher variability of measurements [15]. Thus, we strictly observed stabilization settings.

Apart from the most commonly used parameter, PT, isokinetic dynamometry offers also other parameters such as W [5]. W depends not only on torque development, but also on the range of motion [27] and therefore provides additional information about muscle performance that is especially useful in clinically oriented situations, e.g., therapy/training intervention progress detection [5,28]. For ankle DF, predominantly in eccentric mode of testing, we have observed lower ICC values for W (0.71–0.88) in comparison with PT (0.79–0.98). For ankle PF we have not observed any clear trend of difference in ICC values between PT and W. Similar findings were reported in the study by Woodson et al. [10], where test-retest ICC values for ankle DF in both velocities (30 and 120°/s) pointed to higher variability in W (0.75–0.88) when compared to PT (0.88–0.90). Hölmback et al. [11] also confirmed these results for test-retest ICC at a lower velocity of 30°/s (W: 0.88 and PT: 0.91). It might be assumed that ankle DF in comparison with ankle PF shows higher variability with regard to the range of motion, regardless of the knee joint angle during testing.

Reliability can be determined by how well a measurement maintains its value relative to a sample of repeated measurements (relative reliability, ICC) and/or by the degree to which the repeated measures vary (absolute reliability, SEM) [4]. Even though ICC values for ankle PF/DF and INV/EV isokinetic strength measurements in our study reached predominantly values over 0.77, the magnitude of SEM has to be taken into account to determine whether measurements of a patient, as a part of a treatment or therapy, represent a real change, or whether the measurements are within the natural variability of the measured variables [4]. SEM values for ankle PF have not been reported in earlier studies in healthy adult subjects [6,9,10,18]. The SEM values for concentric PT of ankle DF observed in our study are lower than 2.01 Nm (30°/s) and 2.16 Nm (120°/s) reported in healthy young subjects [11]. Hölmback et al. [11] also confirmed these results for concentric W. SEM values in our study with regard to PT of ankle INV/EV movement were similar to 2.37–2.88 [12] or lower than 3.71–6.92 [8] for INV, and lower than 3.33–6.38 for EV [8,12]. In our study, there was no consistent pattern of differences between actual absolute values of PT between ankle INV and EV, and SEM values for INV and EV were comparable. This is contradictory to findings reported earlier in studies on muscle strength dominance in ankle INV [7,8,26].

It should be noted that the lack of randomization of subject selection should be contemplated when applying the results of the present study to the general population. Also, the small number of participants in a testing sample might be another study limitation that can possibly affect the interpretation of the results for the general population. However, the size of the testing sample is comparable to the ones reported by other research papers on reliability in healthy adults [7,10]. Also, it should be borne in mind that the observed findings can be different from earlier findings reported for specific subjects, such as the elderly [17,29] or children [16,19]. Another limitation of the current study might be that only one testing velocity and the concentric mode of action was used for measurement of ankle INV/EV. However, during pilot testing, not included in this study, most of the subjects reported difficulties or discomfort when generating maximal torque in the medio–lateral direction at higher velocities or in the eccentric mode of action, leading to irrelevant results. Therefore, we decided not to include the eccentric mode or add a higher velocity in the testing protocol.

## 5. Conclusions

The results demonstrated that standardized isokinetic measurements of ankle-related movements in reciprocal PF/DF and INV/EV muscle actions conducted on the IsoMed 2000 isokinetic dynamometer demonstrated ICC ranging from 0.77 to 0.98. Lower ICC values were found for PT in the concentric mode in ankle INV and W in the eccentric mode in ankle DF (120°/s) on the preferred limb, where ICC ranged from 0.64 to 0.71, respectively. These movements might therefore deserve more thorough familiarization with the particular testing conditions. A proper warmup and strict adherence to the testing protocol instructions might increase the reliability of any assessment method and the credibility of the research findings.

## Figures and Tables

**Table 1:** Descriptive statistics and reliability of concentric ankle PF/DF measurements at 30°/s (*n* = 19).

Movement	Variable	Limb	Mean ± SD	ICC(2,1)	SEM	SEM%
Dorsal flexion	PT (Nm)	P	28.0 ± 6.9	0.95	1.5	5.5
		NP	33.2 ± 7.9	0.97	1.4	4.1
		Mean	30.6 ± 7.1	0.97	1.2	4.0
	W (J)	P	14.6 ± 3.5	0.86	1.3	9.1
		NP	17.4 ± 4.2	0.94	1.0	5.9
		Mean	16.0 ± 3.7	0.92	1.0	6.5
Plantar flexion	PT (Nm)	P	113.0 ± 30.2	0.96	6.0	5.3
		NP	115.9 ± 27.0	0.93	7.1	6.2
		Mean	114.4 ± 27.2	0.97	4.7	4.1
	W (J)	P	53.3 ± 14.5	0.92	2.5	4.7
		NP	55.6 ± 12.7	0.87	4.6	8.2
		Mean	54.5 ± 12.8	0.93	3.4	6.2

P—preferred limb, NP—nonpreferred limb, PT—peak torque, W—work, SD—standard deviation, ICC(2,1)—intraclass correlation coefficient, SEM—standard error of measurement, SEM%—relative standard error of measurement.

**Table 2:** Descriptive statistics and reliability of concentric ankle PF/DF measurements at 120°/s (*n* = 18).

Movement	Variable	Limb	Mean ± SD	ICC(2,1)	SEM	SEM%
Dorsal flexion	PT (Nm)	P	19.3 ± 6.2	0.95	1.4	7.2
		NP	23.0 ± 6.6	0.94	1.6	7.0
		Mean	21.1 ± 6.0	0.95	1.3	6.4
	W (J)	P	7.0 ± 3.1	0.92	0.9	12.4
		NP	8.8 ± 3.3	0.91	1.0	11.2
		Mean	7.9 ± 3.0	0.93	0.8	10.0
Plantar flexion	PT (Nm)	P	65.1 ± 17.3	0.85	6.7	10.3
		NP	67.0 ± 17.0	0.87	6.1	9.1
		Mean	66.0 ± 16.1	0.91	4.8	7.3
	W (J)	P	30.4 ± 8.7	0.92	2.4	8.0
		NP	31.6 ± 8.9	0.91	2.7	8.4
		Mean	31.0 ± 8.4	0.94	2.1	6.6

P—preferred limb, NP—nonpreferred limb, PT—peak torque, W—work, SD—standard deviation, ICC(2,1)—intraclass correlation coefficient, SEM—standard error of measurement, SEM%—relative standard error of measurement.

**Table 3:** Descriptive statistics and reliability of eccentric ankle PF/DF measurements at 30°/s (*n* = 20).

Movement	Variable	Limb	Mean ± SD	ICC(2,1)	SEM	SEM%
Dorsal flexion	PT (Nm)	P	51.8 ± 11.8	0.98	1.7	3.2
		NP	51.6 ± 10.9	0.92	3.1	6.0
		Mean	51.7 ± 11.0	0.98	1.6	3.0
	W (J)	P	34.1 ± 8.3	0.79	3.8	11.2
		NP	34.1 ± 8.4	0.88	2.9	8.5
		Mean	34.1 ± 7.7	0.91	2.3	6.8
Plantar flexion	PT (Nm)	P	178.3 ± 75.6	0.89	25.1	14.1
		NP	177.4 ± 69.6	0.95	15.6	8.8
		Mean	177.8 ± 69.7	0.95	15.6	8.8
	W (J)	P	78.9 ± 30.0	0.92	8.5	10.8
		NP	79.2 ± 26.1	0.92	7.4	9.3
		Mean	79.1 ± 26.9	0.96	5.4	6.8

P—preferred limb, NP—nonpreferred limb, PT—peak torque, W—work, SD—standard deviation, ICC(2,1)—intraclass correlation coefficient, SEM—standard error of measurement, SEM%—relative standard error of measurement.

**Table 4:** Descriptive statistics and reliability of eccentric ankle PF/DF measurements at 120°/s (*n* = 19).

Movement	Variable	Limb	Mean ± SD	ICC(2,1)	SEM	SEM%
Dorsal flexion	PT (Nm)	P	54.8 ± 11.8	0.79	5.4	9.8
		NP	55.3 ± 10.4	0.91	3.1	5.6
		Mean	55.0 ± 10.5	0.91	3.1	5.7
	W (J)	P	28.5 ± 6.5	0.71	3.5	12.2
		NP	28.6 ± 6.3	0.83	2.6	9.1
		Mean	28.6 ± 5.7	0.85	2.2	7.7
Plantar flexion	PT (Nm)	P	154.8 ± 55.3	0.86	20.7	13.4
		NP	155.4 ± 47.1	0.95	10.5	6.8
		Mean	155.1 ± 49.7	0.95	11.1	7.2
	W (J)	P	52.2 ± 17.3	0.79	7.9	15.2
		NP	54.0 ± 14.6	0.92	4.1	7.7
		Mean	53.1 ± 15.2	0.93	4.0	7.6

P—preferred limb, NP—nonpreferred limb, PT—peak torque, W—work, SD—standard deviation, ICC(2,1)—intraclass correlation coefficient, SEM—standard error of measurement, SEM%—relative standard error of measurement.

**Table 5:** Descriptive statistics and reliability of concentric ankle INV/EV measurements at 30°/s (*n* = 20).

Movement	Variable	Limb	Mean ± SD	ICC(2,1)	SEM	SEM%
Eversion	PT (Nm)	P	26.6 ± 7.4	0.92	2.1	7.9
		NP	24.7 ± 6.9	0.90	2.2	8.8
		Mean	25.7 ± 6.4	0.94	1.6	6.1
	W (J)	P	11.9 ± 3.7	0.94	0.9	7.7
		NP	10.7 ± 2.8	0.87	1.0	9.5
		Mean	11.3 ± 3.0	0.94	0.7	6.6
Inversion	PT (Nm)	P	22.7 ± 5.5	0.64	3.3	14.5
		NP	24.2 ± 6.1	0.89	2.0	8.4
		Mean	23.5 ± 5.0	0.83	2.1	8.8
	W (J)	P	9.9 ± 2.3	0.77	1.1	11.2
		NP	10.8 ± 3.0	0.90	1.0	8.8
		Mean	10.3 ± 2.4	0.88	0.8	7.9

P—preferred limb, NP—nonpreferred limb, PT—peak torque, W—work, SD—standard deviation, ICC(2,1)—intraclass correlation coefficient, SEM—standard error of measurement, SEM%—relative standard error of measurement.
